# Reliability and validity of the Turkish version of the Diabetes Distress Scale for type 2 diabetes and distress levels of the participants

**DOI:** 10.3906/sag-1903-121

**Published:** 2020-04-09

**Authors:** Özge TELCİ ÇAKLILI, Güneş ALKAYA FEYİZOĞLU, Selcan TÜLÜ, Nazlı DİZMAN, İrem Sıdıka BOZKURT, Aytekin OĞUZ

**Affiliations:** 1 Kocaeli State Hospital, Kocaeli Turkey; 2 Department of Internal Medicine, Faculty of Medicine, İstanbul Medeniyet University, İstanbul Turkey

**Keywords:** Diabetes Distress Scale, Turkish, validation, type 2 diabetes

## Abstract

**Background/aim:**

Studies have shown an increased depression rate in patients with type 2 diabetes mellitus (T2DM) compared to the normal population. It is now acknowledged that patients suffer from distress rather than depression. Our aim was to validate the Turkish version of the Diabetes Distress Scale (DDS) and to show distress levels of the participants.

**Materials and methods:**

The scale was translated from English to Turkish by the authors and translated back to English. Between August 2015 and January 2016 all the patients who were referred to the T2DM Clinic of İstanbul Medeniyet University were screened, and eligible patients were recruited. For calculating internal consistency Cronbach’s alpha coefficient was used.

**Results:**

A total of 205 patients [120 females (58.5%), 85 males (41.5%)] were included. Cronbach’s alpha coefficient was 0.874, showing internal consistency. The Spearman Brown correlation coefficient was calculated between the first 9 and second 8 questions as 0.884. Thetotal variances were explained at a level of 66.2% with 4 factors. Sixty-three patients (30%) had a score of ≥3, indicating diabetic distress. Correlation analysis showed a significant correlation between total score and HbA1c levels (r = 0.152 and P = 0.038).

**Conclusion:**

The Turkish version of the DDS for type 2 diabetes is a reliable tool for assessment of distress levels.

## 1. Introduction

Type 2 diabetes mellitus (T2DM) is a growing epidemic and it is projected that by 2035 almost 600 million people in the world will have the disease [1]. This increase in T2DM prevalence has made T2DM one of the major health issues of the world in the last decade. From cardiovascular diseases to end-stage kidney disease, T2DM causes morbidity and mortality second to none [2].

In recent years, along with metabolic complications, psychological damage caused by T2DM diagnosis has also been investigated [3]. Studies have shown increased depression rates in patients with T2DM compared to the normal population [4] and an increased mortality risk is also associated with depression in patients with T2DM [5]. Although depression should still be suspected in patients with prominent symptoms, it is now acknowledged that patients suffer from T2DM-associated distress rather than depression [6,7]. Major depressive disorder is diagnosed with the presence of at least 5 of the 9 symptoms that persist for at least 2 weeks [8]. It is not diagnosed according to a specific etiology. However, distress consists of the worries, concerns, and fears of individuals and it has a broader spectrum of feelings [7]. Three-dimensional study has shown that 84% of patients with moderate-high distress did not reach a diagnosis of major depressive disorder [9]. Patients with diabetes and positive depression scores do not always have the symptoms of major depressive disorder to reach a diagnosis [10]. Within this context, a more target-specific tool is needed to assess distress of patients with diabetes. Otherwise, patients would be misdiagnosed with depression and treated pharmacologically although all they suffer from is diabetes-related anxiety.

The Diabetes Distress Scale (DDS) was created by Polonsky et al. [11] to diagnose T2DM-associated distress. It is a 17-item psychological measurement tool that uses a Likert scale with each item scored from 1 (no distress) to 6 (serious distress) to reflect distress experienced over the last month. The DDS examines patients’ distress regarding their treatment, overall life style behavior, social support, and relationship with healthcare providers. There are four sources of distress identified by the scale: regimen distress, emotional burden, interpersonal distress, and physician distress. This study aims to validate the Turkish version of the DDS and assess the DDS scores of patients with T2DM.

## 2. Material and methods

### 2.1. Ethics

Ethical approval for the study was obtained from the İstanbul Medeniyet University Ethics Committee (Date: 07.29.2015, Decision Number: 2015/0107). Patients were given consent forms to sign and the rules of the Helsinki Declaration were followed throughout the study.

### 2.2. Permission

Permission to use and translate the scale was obtained from the creator of the scale, William Polonsky, via email. The Turkish version of the scale was also shared with the author.

### 2.3. Translation

The scale was translated from English to Turkish by the authors (ÖTÇ, AO, ND) and then retranslated (back-translation) to English by the Head of the Foreign Languages Department of İstanbul Medeniyet University. Three internists evaluated and gave scores to assess the context validity of the translation.

### 2.4. Sample size calculation

For validation studies, the general approach is to recruit 5–10 subjects for each question that the scale possesses [12]. A 20% loss was presumed and 205 patients were included. 

### 2.5. Participants

Between August 2015 and January 2016 all the patients who were referred to the T2DM Clinic of İstanbul Medeniyet University’s Department of Internal Medicine were screened. Patients were included in the study if they had a T2DM diagnosis and gave consent to participate in the study. Exclusion criteria were being ≤18 years old, having type 1 diabetes, presence of a psychological disease, presence of a morbidity that can affect life quality (cancer, chronic kidney disease etc.), being pregnant, and not having sufficient intellectual capacity to understand the questions of the scale. The DDS was applied to illiterate patients with the help of their relatives.

Patients’ demographic characteristics including age, sex, T2DM duration, educational status, medications, body mass index (BMI), waist circumference, and metabolic parameters (fasting glucose level, HbA1c, etc.) were recorded. There were no newly diagnosed drug-naive patients, the mean duration of diabetes was 10.7 years, and 56.9% of the patients (n = 116) were on insulin treatment.

### 2.6. Assessment of distress

The DDS is a 17-item psychological measurement tool that uses a Likert scale with each item scored from 1 (no distress) to 6 (serious distress) to reflect distress experienced over the last month. The total score is calculated by dividing the sum of the answers by 17. A score of ≥3 is defined as T2DM-related distress. There are four subscales and subscale scores are also calculated by dividing the sum of subscale answers to the total question count of the subscale. Subscales are as follows: the emotional burden subscale with 5 questions, the physician-related distress subscale with 4 questions, the regimen-related distress subscale with 5 questions, and the interpersonal distress subscale with 3 questions. The Turkish translation of the scale can be found at http://behavioraldiabetes.org/scales-and-measures/ and in Table 1.

**Table 1:** Turkish version of Diabetes Distress Scale.

Sorunlar	Sorun değil	Hafif bir sorun	Orta düzeyde sorun	Aslında ciddi olabilecek bir sorun	Ciddi sorun	Çok ciddi sorun
1. Diyabetin her gün zihinsel ve fiziksel enerjimi çok fazla aldığını hissediyorum.	1	2	3	4	5	6
2. Doktorumun diyabet ve diyabet bakımı konusunda yeterli bilgiye sahip olmadığını hissediyorum.	1	2	3	4	5	6
3. Diyabet ile yaşamı düşündüğüm zaman kızgın, endişeli ve / veya depresif hissediyorum.	1	2	3	4	5	6
4. Doktorumun diyabetimi yönetmede beni yeterince açık yönlendirmediğini hissediyorum.	1	2	3	4	5	6
5. Kan şekeri takibimi yeterli sıklıkta yapmadığımı hissediyorum.	1	2	3	4	5	6
6. Diyabet rutinimde sıklıkla başarısız olduğumu hissediyorum.	1	2	3	4	5	6
7. Arkadaşlarımın veya ailemin öz - bakım çalışmalarımı yeterince desteklemediğini hissediyorum.(örneğin: ‘yanlış’ yiyecekler yemem için teşvik etmeleri, planladıkları aktivitelerin benim programımla uyuşmaması)	1	2	3	4	5	6
8. Diyabetin hayatımı kontrol ettiğini hissediyorum.	1	2	3	4	5	6
9. Doktorumun endişelerimi yeterince ciddiye almadığını hissediyorum.	1	2	3	4	5	6
10. Diyabetimi gün be gün yönetebileceğimden emin hissetmiyorum.	1	2	3	4	5	6
11. Ne yaparsam yapayım sonunda ciddi uzun dönem komplikasyonların olacağını hissediyorum.	1	2	3	4	5	6
12. İyi bir yeme planına yeteri kadar bağlanmadığımı hissediyorum.	1	2	3	4	5	6
13. Arkadaşlarım ve ailemin diyabetle yaşayabilmenin ne kadar zor olduğunu anlamadığını hissediyorum.	1	2	3	4	5	6
14. Diyabetle yaşamın gerektirdiklerinden bunalmış hissediyorum.	1	2	3	4	5	6
15. Diyabetim için düzenli olarak görüşebileceğim bir doktora sahip olmadığımı hissediyorum.	1	2	3	4	5	6
16. Kendi diyabet takibimi yapabilecek kadar motivasyon hissetmiyorum.	1	2	3	4	5	6
17. Arkadaşlarımın ve ailemin istediğim duygusal desteği verdiğini hissetmiyorum.	1	2	3	4	5	6

Not: Diyabet yerine şeker hastalığı tanımı da kullanılabilir.

### 2.7. Statistical analysis

In addition to the descriptive statistical methods (mean, standard deviation, median, frequency, percentage, minimum, and maximum) for comparison of the quantitative data, the Student t-test was used for two-group comparisons of the variables with normal distribution and the Mann–Whitney U test was used for two-group comparisons of the variables with nonnormal distribution. One-way ANOVA was used to compare three or more groups for variables with normal distribution and the Kruskal–Wallis test was used to compare three or more groups with nonnormal distribution. Pearson correlation and Spearman correlation coefficients were used to evaluate the relationships between the variables. Significance level was set at P < 0.01 and P < 0.05.

Cronbach’s alpha was calculated to check for internal consistency of the scale. The split-half method with Spearman Brown correlation coefficient was used to assess intraclass consistency. Exploratory factor analysis was used to evaluate the construct validity of the questionnaire. Before factor analysis, some preliminary tests were used. The Kaiser–Meyer–Olkin (KMO) test was used to assess efficiency of the sample size. The Bartlett test of sphericity was used to evaluate whether the diagonal terms of the correlation matrix were 1 and the nondiagonal terms were 0. Principal component analysis was used to reveal the structure of factors. A rotation component matrix was used to determine the factor structures. Factors were determined by grouping the questions according to those with high weights on factors. Analyses were performed with SPSS 21 (IBM Corp., USA).

## 3. Results

A total of 205 patients [120 females (58.5%), 85 males (41.5%)] were included. Demographic features of the patients are listed in Table 2.

**Table 2:** Demographic characteristics of the cohort.

Variables	Patients (n = 205)
Age (years)	55.29 ± 10
Diabetes duration (years)	10.7 ± 6.8
Waist circumference (cm)	Males: 103 ± 12
	Females: 108 ± 12
Body mass index (kg/m2 )	32.2 ± 12
Fasting plasma glucose (mg/dL)	184 ± 89
Hemoglobin A1c (%)	8.5 ± 2.2
Creatinine (mg/dL)	0.8 ± 0.2
Education status	N %Illiterate 22 10.7Elementary school 104 50.7Middle school 18 8.8High school 32 15.6University 29 14.1

Cronbach’s alpha coefficient was found to be 0.874, showing internal consistency. The Spearman Brown correlation coefficient was calculated between the first 9 and second 8 questions as 0.884. This result shows that the intraclass consistency of the questionnaire was good. Exploratory factor analysis was used to evaluate the construct validity of the questionnaire. The KMO parameter was calculated as 0.853. This result shows that the sample size was appropriate for factor analysis. The Bartlett test of sphericity was used to evaluate whether the diagonal terms of the correlation matrix were 1 and the nondiagonal terms were 0. In our study, we cannot reject the null hypothesis (population correlation matrix is an identity matrix) (P < 0.001). The diagonal terms of the antiimage correlation matrix vary from 0.792 to 0.932. Principal component analysis was used to reveal the structure of factors. Explained total variances are shown in Table 3. The total variances were explained at a level of 66.2% with 4 factors.

**Table 3:** Explained total variance.

Factor	Eigenvalues			Rotation sums of squared loadings		
	Total	Variance %	Cumulative %	Total	Variance %	Cumulative %
1	5.741	33.772	33.772	3.505	20.616	20.616
2	2.303	13.545	47.316	2.909	17.114	37.730
3	1.916	11.273	58.589	2.491	14.654	52.383
4	1.287	7.571	66.161	2.342	13.778	66.161
5	0.806	4.744	70.904			
6	0.674	3.964	74.868			
7	0.621	3.651	78.520			
8	0.552	3.245	81.764			
9	0.527	3.102	84.866			
10	0.470	2.764	87.630			
11	0.450	2.646	90.276			
12	0.357	2.102	92.378			
13	0.314	1.849	94.226			
14	0.284	1.670	95.896			
15	0.274	1.611	97.507			
16	0.231	1.361	98.868			
17	0.192	1.132	100.000			

A rotation component matrix was used to determine the factor structures (Table 4). Factors were determined by grouping the questions with high weights on factors. In our study, weights of ≥0.48 were thought of as sufficient to include as a factor. Those factors were developed like the original scale. None of the names of the factors were revised. The only difference between the original scale and the validated scale was the 16th question, which was seen for factor 1.

**Table 4:** Rotational factor matrix.

Factor				
	1	2	3	4
Question 1	0.710	0.152	0.019	–0.024
Question 2	0.096	0.873	0.034	0.004
Question 3	0.752	0.082	0.065	0.072
Question 4	0.152	0.875	0.104	0.046
Question 5	0.031	0.003	0.094	0.771
Question 6	0.269	0.120	0.062	0.787
Question 7	0.138	0.093	0.869	0.026
Question 8	0.655	0.087	0.228	0.270
Question 9	0.092	0.870	0.145	–0.028
Question 10	0.676	0.033	0.082	0.422
Question 11	0.616	0.115	0.112	0.284
Question 12	0.326	0.065	0.043	0.686
Question 13	0.171	0.045	0.877	0.058
Question 14	0.790	0.080	0.206	0.121
Question 15	0.146	0.667	0.005	0.341
Question 16	0.482	0.279	0.224	0.394
Question 17	0.155	0.123	0.867	0.166

Mean diabetes duration of the population was 10.2 ± 6.9 years and 57.1% of the patients were on insulin treatment. Table 2 shows patients’ education statuses. The mean total score of the patients was 2.5 ± 0.9. Sixty-three patients (30%) had a score of ≥3, indicating diabetic distress. Female patients had higher total scores than male patients (P = 0.002). They also had higher scores in the emotional burden, regimen-related distress, and interpersonal distress subscales (P = 0.003, P = 0.01, and P = 0.004, respectively). A significant difference in total score and scores of ≥3 was observed between patients who used insulin and who did not (P = 0.01 and 0.02, respectively), with higher scores in the insulin group (Table 5). There was a statistically significant difference between patients with HbA1c levels of ≥9 (75 mmol/mol) and <7 (53 mmol/mol) in total distress and the emotional burden subscale, both being higher in the high HbA1c group (P=0.02 and P=0.01, respectively).

**Table 5:** Comparison of patients on insulin with patients on oral antidiabetics.

	Insulin users	Non-insulin users	P-value
Age	55.59 ± 10.81	54.67 ± 9.66	0.53
Duration	12.75 ± 7.19	8.07 ± 5.44	<0.00
Waist	106.93 ± 12.71	104.86 ± 12.51	0.24
Length	161.55 ± 11.90	163.38 ± 9.49	0.24
Weight	83.61 ± 16.02	84.80 ± 14.96	0.59
Glucose	203.84 ± 98.94	176.89 ± 109.11	0.04
Urea	31.12 ± 10.51	30.34 ± 9.81	0.61
Creatinine	0.83 ± 0.21	0.82 ± 0.16	0.72
Cholesterol	196.51 ± 48.77	198.20 ± 40.38	0.85
Triglyceride	189.95 ± 126.53	223.69 ± 159.69	0.36
HDL	43.02 ± 14.54	40.84 ± 11.19	0.38
LDL	120.70 ± 39.63	119.27 ± 30.63	0.84
HbA1c	9.44 ± 2.98	7.95 ± 2.12	0.003
Emotional	3.56 ± 1.37	2.86 ± 1.39	0.009
Physician	1.89 ± 1.23	1.59 ± 1.07	0.10
Regimen	2.90 ± 1.26	2.77 ± 1.22	0.54
Interpersonal	2.26 ± 1.70	2.02 ± 1.50	0.38
Sum	2.74 ± 0.99	2.39 ± 1.00	0.01

Correlation analysis showed that there was a significant correlation between total score and HbA1c levels (r = 0.152 and P = 0.038), and also between the emotional burden distress subscale and BMI (r = 0.166 and P = 0.01).

## 4. Discussion

In this study reliability and validation analysis of the Turkish DDS was performed. The analysis showed good consistency within scale questions. Also, intraclass consistency was observed. Compatible with the original scale, our study also showed four factors. The sixteenth question of the scale was under the subgroup of “treatment-related distress” in original scale whereas, it was found in the “emotional burden” subscale of our study. Similarly, in the validation analysis of the Norwegian translation of the scale, the 11th question was found in “treatment-related distress” instead of “emotional burden-associated distress” [13]. These differences are often a result of cultural variances and therefore these differences underline the need for a validation analysis of the assessment of scale questions. 

Another important issue to consider is the population the scale is applied to. In our study population, half of the patients were elementary school graduates and 10% of the subjects were illiterate. The Turkish population between 50 and 60 years is 5% illiterate and 60% of the individuals are elementary school graduates according to 2014 data of the Turkish Statistical Institute (www.tuik.gov.tr), which is similar to our cohort. Therefore, it can be said that our cohort represents the Turkish population.

Erkin et al. translated the DDS to Turkish and found it valid and reliable [14]. However, they included patients with type 1 diabetes although there is a different distress scale for these patients [15]. The distress of patients with type 1 diabetes has a different aspect. Although the DDS for type 2 diabetes has four major categories (regimes distress, emotional burden, interpersonal distress, and physician distress), the DDS for type 1 diabetes has seven. These include powerlessness, eating distress, management distress, hypoglycemia distress, negative social perceptions, physician distress, and friend/family distress. There are some overlapping items, but eating distress and hypoglycemia distress significantly differ from the concerns of patients with T2DM. It is also reported that distress levels are higher in younger patients with type 1 diabetes, a finding we did not see in our cohort with T2DM. Inclusion of patients with type 1 diabetes would also alter mean BMI, waist circumference, and age values, disturbing the overall analysis. Moreover, compared to the work of Erkin et al., our study was conducted with a larger population.

Glycemic control is an important factor that triggers stress in patients with T2DM [16]. Patients with increased distress also have high HbA1c levels. Similarly, this association was observed in our correlation analysis. The link between glycemic control and psychological health has a bidirectional pathway [17,18]. It is a vicious cycle to break, but some reports were published with promising results investigating treatment modalities to improve glycemic control by reducing distress [19].

Another factor that has been observed is the emotional burden that BMI has for these patients. Physical appearance has always been an important factor of psychological well-being [20]. Thus, it is no surprise that high BMI was correlated with increased distress levels of the patients, especially on an emotional level. Studies have shown that people sometimes rank themselves in society according to their weight. In the modern world, obesity is not only a major somatic health burden; it is also a psychological cause of distress [21]. Females have always been affected by body changes more than men [22], and our study results show that women have higher distress levels than men. This difference may be attributed to the aforementioned appearance-related issues.

Insulin treatment is often an inconvenient choice for patients [23]. Patients who start insulin treatment often have side problems like visual limitations or multidrug use, and along with these issues injection fears and the strict rules of the treatment (punctual injection time,

dose adherence with glucose monitoring, cold-chain transportation, etc.) create patients’ reluctance. In our study, patients who were on insulin treatment showed more distress than patients who were not. It is fair to say that initiation of insulin is another factor for distress, which can increase the discomfort patients already feel about the disease. As expected, patients receiving insulin treatment had longer disease duration and poor glycemic control compared to patients not on insulin.

In conclusion, the Turkish version of the DDS for T2DM is a reliable tool for assessment of distress levels in this population. This study shows that distress is linked to insulin use, high HbA1c levels, and high BMI, suggesting a solid interaction between poor glycemic control and psychological health. This underlines the fact that treatment of T2DM with weight loss is the best way to lessen the psychological discomfort patients have.

## Acknowledgments

The authors would like to thank Elif Kır (Head of the Foreign Language School of İstanbul Medeniyet University) for her work translating the questionnaire from Turkish to English.
